# The Mechanisms of Medial Pedicle Wall Violation: Insertion Method Is as Important as Correct Cannulation of the Pedicle

**DOI:** 10.1155/2014/283783

**Published:** 2014-10-21

**Authors:** Cengiz Isik, Kamil Cagri Kose, Mustafa Erkan Inanmaz, Suleyman Murat Tagil, Hakan Sarman

**Affiliations:** ^1^Department of Orthopedics and Traumatology, Abant Izzet Baysal University Medical School, Golkoy, 14280 Bolu, Turkey; ^2^Department of Orthopedics and Traumatology, Marmara University Medical School, Istanbul, Turkey; ^3^Department of Orthopedics and Traumatology, Sakarya University Medical School, Sakarya, Turkey; ^4^Department of Anatomy, Turgut Özal University, Medical School, Ankara, Turkey

## Abstract

A cadaver study aims to determine the mechanisms of medial pedicle wall violation after a correct cannulation of the pedicle. The study presents finding out the effect of insertion angle and insertion force on medial wall violation. We used 100 lumbar pedicles of cadavers. Special wooden blocks were produced to simulate a fixed angle fault after a correct pedicle cannulation. Pedicles were divided into 4 groups: 10-degree free drive (group 10), 15-degree free drive (group 15), 10-degree push drive (group 10P), and 15-degree push drive (group 15P). After insertion of pedicle screws, laminectomies were done and the pedicles were evaluated from the inside. Pedicle complications were more in group 10P than group 10 (*P* = 0.009). Medial wall fracture (*P* = 0.002) and canal penetration were more in group 15P than group 15 (*P* = 0.001). Groups 10P and 15P were similar regarding medial wall fractures but canal penetration was significantly higher in group 15P (*P* = 0.001). Medial wall breaches can happen after correct cannulation of pedicles. Change in insertion angle is one factor but the most important factor is the use of a pushing force while inserting a screw. The pedicle seems to be extremely tolerant to insertion angulation mistakes up to 10 degrees and tends to lead the screw into the correct path spontaneously.

## 1. Introduction

Transpedicular instrumentation is nowadays the standard fixation method for the treatment of various disorders of the spine [1, 2]. Screw fixation is superior to sublaminar wires and hooks especially in fracture, listhesis, and deformity patients where the anchorage power and load bearing capacity of the implant is important. However pedicle screws are not complication-free.

Some complications of pedicle screws are (a) malposition of the screw (medial wall breach, intraforaminal placement, and sacroiliac joint violation), (b) fracture of the pedicle, (c) injury to the cord or nerve roots, and (d) fracture of the implant [3–8]. To prevent these complications it is mandatory to apply the screws with the correct technique and in the direction of the pedicle. Several navigation systems, robots, and hand tools have been developed to enable correct cannulation of the pedicles [5, 6, 9–14]. Unfortunately these systems are not error-free. They also have a learning curve; most of them are expensive and time consuming and these disadvantages limit their widespread use. Because of these entire reasons freehand pedicle screw placement is still the most frequent method of pedicle screw placement [3–8]. Finding the appropriate entry point and then determining the correct mediolateral and craniocaudal angles are important in transpedicular fixation [15, 16]. After pedicle cannulation, ball tipped probes are used to control the walls and the screws are then placed into the pedicles. Between probe control and placement of the pedicle screws the surgeon can change the angle of insertion unintentionally. Whether this causes any medial pedicle wall violations is unknown. In our practice sometimes although the surgeon is sure that he did not feel anything wrong during ball tipped probe examination, we see that there are pedicle wall breaches in postoperative CT examinations. It is not clear whether the things went wrong during or after pedicle screw cannulation.

In this study we aimed to find out medial wall violating effects of (a) changing the insertion angle of the screw after correct cannulation of the pedicles and (b) changing the downward insertion force.

## 2. Patients and Methods

We used 100 lumbar pedicles of 10 male cadavers for this study. Special wooden blocks were produced to simulate a fixed angle fault after a correct pedicle cannulation.

A wooden block of 2 × 2 × 4 cm was marked in midline. It was drilled with a 3 mm drill vertically. Then using an angle adjustable drill guide, the block was drilled again with 5-, 10-, 15-, and 20-degree angles with a starting point parallel to the first hole.

A second block was produced in the same fashion this time including the 20-degree tunnel as well. Ten dry lumbar vertebrae (20 pedicles) were used to make a preliminary study. In this study we saw that 5-degree insertion fault did not produce any breach at all and that 20-degree insertion fault was nearly impossible in a living person because of the paravertebral muscles and so we removed the 5- and 20-degree tunnels from the experiment protocol.

**Figure 1:**
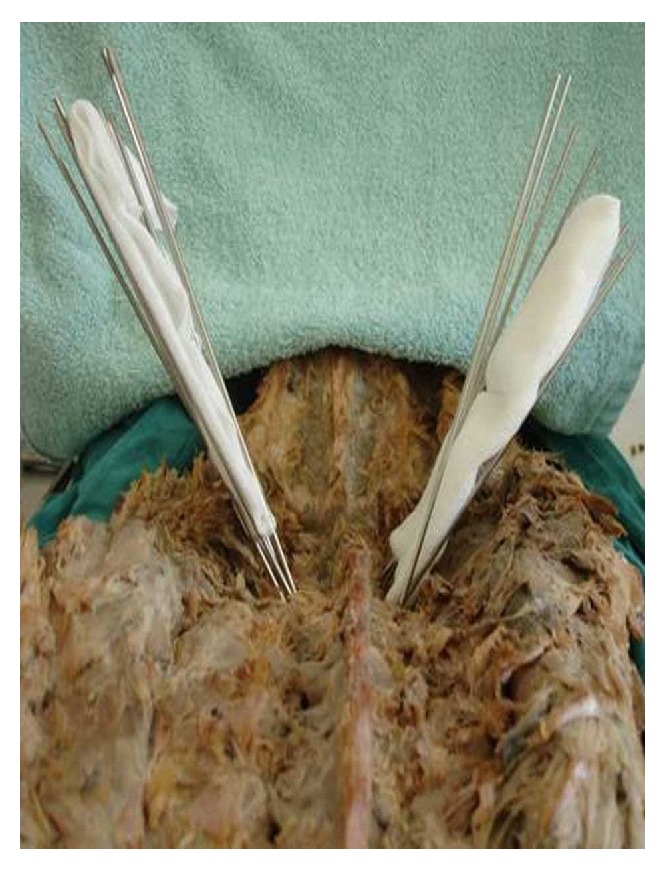
All wires in place. There is a gauze sponge between the 10-degree wires on the right and the 15-degree wires on the left.

**Figure 2:**
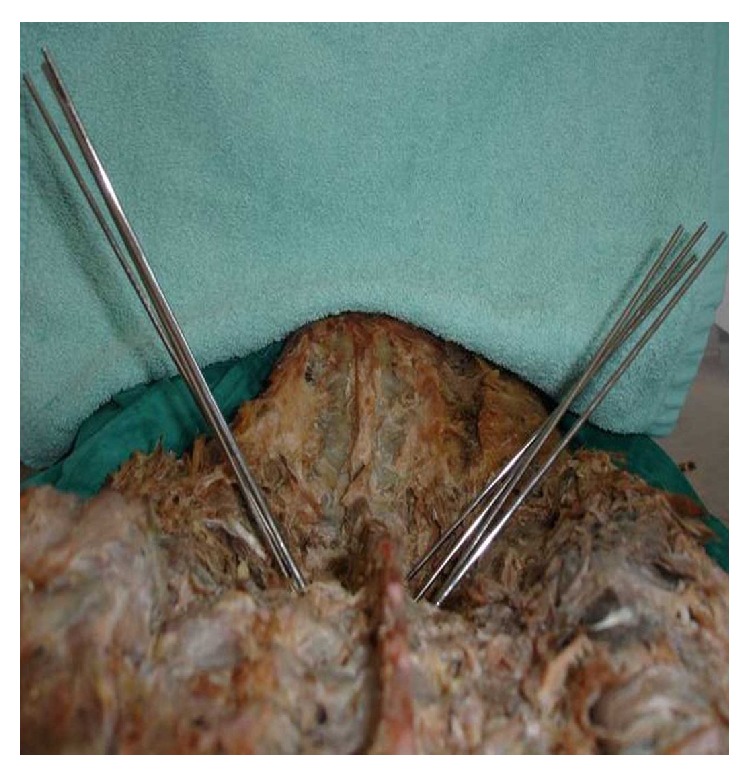
After removal of the primary wires which were in the correct pedicular trajectory. The 10-degree wires are on the right and the 15-degree wires are on the left.

The cadavers were prepared in a standard fashion in prone position. The lumbar spines of the cadavers were exposed through a midline incision. Using the classical landmarks the left and right pedicles of the cadavers were cannulated with a 3 mm pedicle probe. Then a ball tipped probe was used to check the pedicle pathway. All cannulations were done by an experienced spine surgeon and two other surgeons checked each hole. When the walls were found to be intact, a 3 mm K wire was put into the pedicle representing the correct pedicle pathway. Then the wooden block was slid over the wire through the “0” degree hole. The block was positioned parallel to the spinous processes and then a second K wire was introduced using a power drill through the 10- or 15-degree holes (10 degrees on the right and 15 degrees on the left) (Figure 1). They were implanted 3-4 cm deep. After these first the “0”-degree wire and then the block were removed. Now we had a correctly cannulated pedicle and a K wire representing 10- or 15-degree medial deviation from its axis (Figure 2).

**Figure 3:**
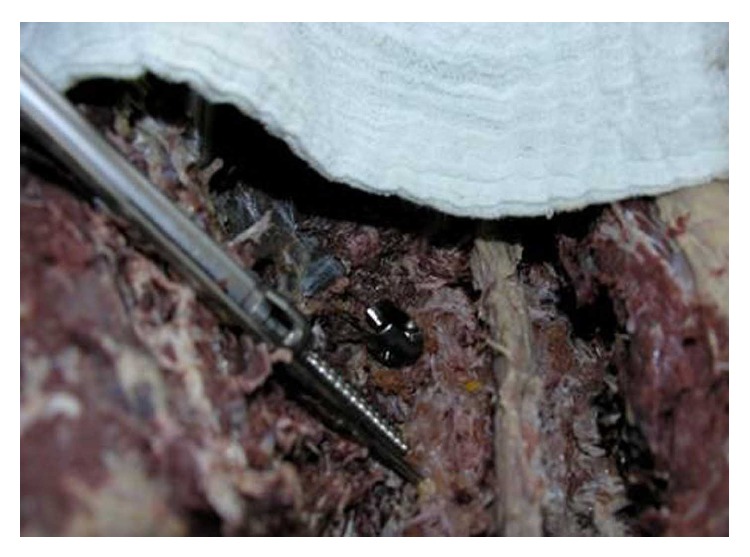
Starting insertion of the screw parallel to the 10-degree wire.

**Figure 4:**
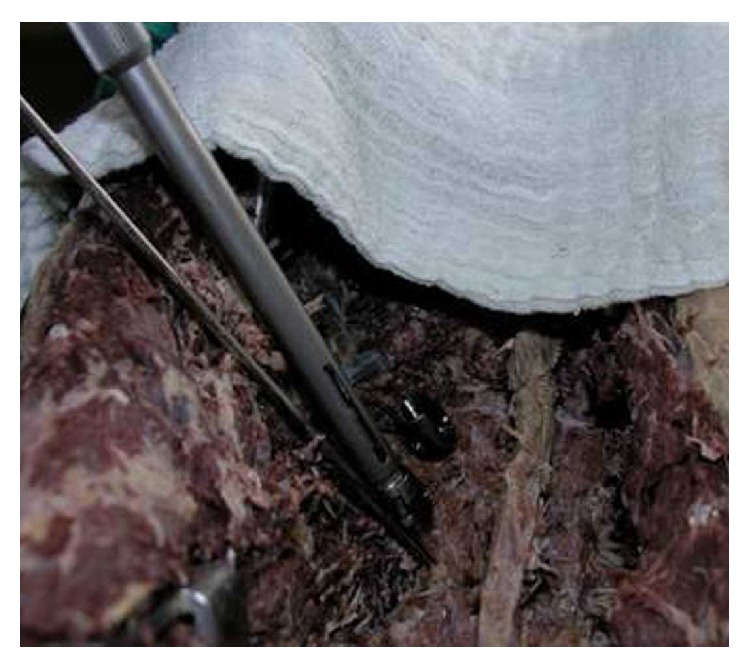
The 10-degree screw is inserted. The change in angle is seen after resistance and change of the trajectory.

**Figure 5:**
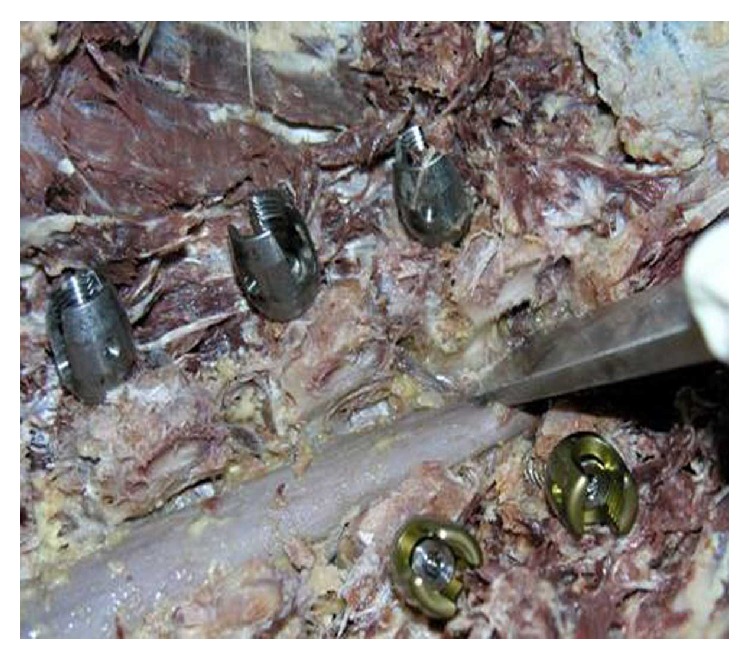
Intact medial pedicle walls after laminectomy following insertion of 10-degree screws without force application.

In the first 5 cadavers, 6 × 45 mm screws (Blackstone-Orthofix Inc., USA) and 6.5 × 45 mm screws (having a self-tapping notch at the tip) (Novel2 varian, South Korea) were used. The screws were inserted parallel to the K wires and the screw drivers were only rotated without any pushing force upon any resistance (Figure 3). When the screw hit a pedicle wall it was left free to either lie on the wall or not advance further or to change its direction itself and continue down the pedicle pathway (Figure 4). After full insertion of the screws, laminectomies were performed to see whether there was any violation of the medial pedicle wall. All screws were photographed during and after screw insertion and laminectomy (Figure 5).

**Figure 6:**
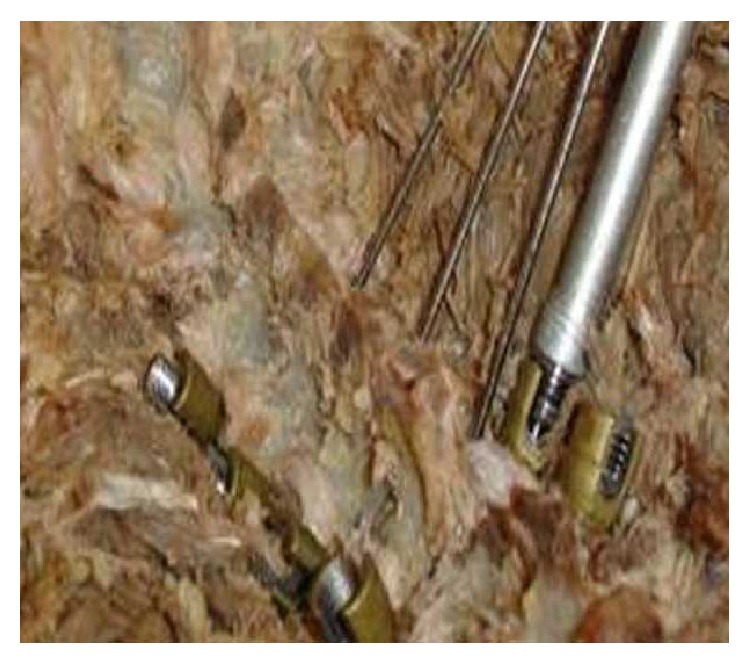
Pedicle screws after forced insertion with 15-degree angle.

**Figure 7:**
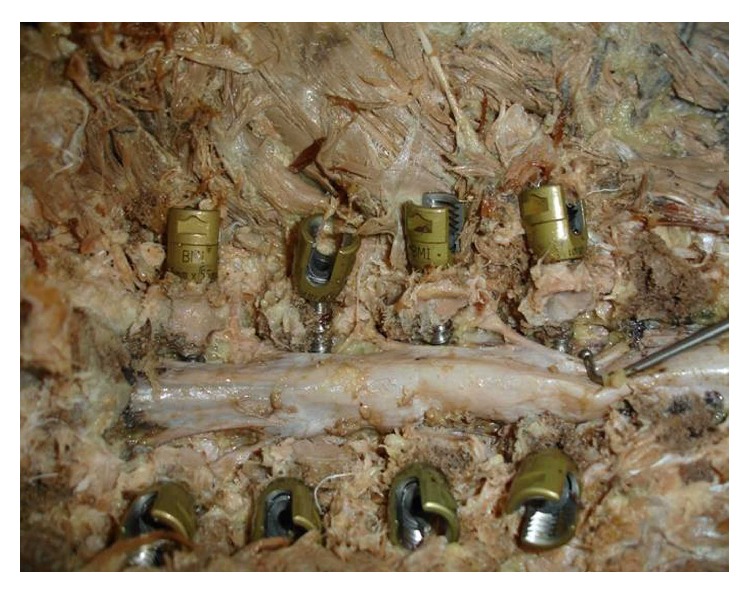
Pedicle walls after laminectomy. 15-degree screws are on the upper side and 10-degree screws are on the lower side.

In the second group of 5 cadavers, again 6 × 45 mm screws (Blackstone-Orthofix Inc., USA) and 6.5 × 45 mm screws having a self-tapping notch at the tip (Novel2 varian, South Korea) were used. This time, the screws were inserted parallel to the K wires and a pushing force was applied to the screw drivers when there was any feeling of resistance (Figure 6). After full insertion of the screws, laminectomies were performed to see whether there was any violation of the medial pedicle wall. All screws were photographed during and after screw insertion and laminectomy (Figure 7).

The findings were evaluated in 4 groups: 10-degree free drive (group 10), 15-degree free drive (group 15), 10-degree push drive (group 10P), and 15-degree push drive (group 15P).

SPSS version 13.0 was used for statistical evaluation (SPSS Inc., USA). Chi-square test was used for analysis. *P* < 0.02 was considered as significant.

## 3. Results

None of the pedicles were excluded. There was a significant difference between group 10 and group 10P regarding pedicle complications (*P* = 0.009). There was also a significant difference between group 15 and group 15P regarding medial wall fracture (*P* = 0.002) and canal penetration (*P* = 0.001). Leaning on the pedicle wall was significantly higher in group 15 (*P* = 0.002). The total number of pedicle related complications was significantly higher in group 15P (*P* = 0.001). When groups 10P and 15P were compared, there was no significant difference regarding medial wall fractures (*P* = 0.225) but canal penetration was significantly higher in group 15P (*P* = 0.001). When group 10 and group 15 were compared, there was no difference regarding medial wall fracture or canal penetration (*P* = 0.312) but leaning on the pedicle wall was significantly higher in group 15 (*P* = 0.002) in Table 1.

Then the whole study group was reanalyzed regarding the presence of pushing while driving the screw into the pedicle. Group 10 and group 15 were regarded as one group and group 10P and group 15P were regarded as another group. It was seen that nearly all pedicle related complications had taken place in the “pushing” groups. The pedicle wall fractures and canal penetrations were significantly more in “pushing” groups (*P* = 0.001 and *P* = 0.001) and medial wall leaning was more in the “free drive” groups (*P* = 0.003). When medial wall leaning was removed from the list of complications, the free drive technique had a complication rate of 2% and the “push drive” technique had a complication rate of 56% in Table 2.

Another analysis was done regarding the pedicle screws. In this analysis medial wall leaning was again among the complication list. The presence of a self-tapping notch on the screw or a screw diameter 0.5 mm thicker did not adversely affect the rate of complications but canal penetration was more frequent in the 15-degree push drive group when self-tapping; 0.5 mm thicker screws were used in Table 3.

When free drive and push drive groups were compared, 96.5% of the complications were seen in push drive groups. The complication rate of free drive groups was 3.5%. Medial wall leaning was only seen in free drive groups. Sixty-five percent of the intact pedicles were in the free drive groups and 35% were in the push drive groups.

## 4. Discussion

Neurological injury is the most feared complication of all in spinal surgery nearing a hundred years old and transpedicular fixation nearing 35 years old. Neurological injury is still possible in the most experienced hands and centers. Although navigation systems have been developed to decrease the complication rates, they are costly and time consuming. That is why they are used in only few centers. The most commonly used guidance method is the use of AP and lateral fluoroscopy and the freehand pedicle drilling/cannulation technique.

Kosay et al. reported that the oil particles originating from the bone marrow coming out of the hole of the pedicle probe are reliable indicators that the pedicle had been drilled correctly [17]. American Back Society had questioned its members about the complications of pedicle screw insertion. Thirteen surgeons used pedicle screws in 617 cases and had 169 complications of which 59 (34.9%) took place during surgery. In addition to this, 32 screw malpositions were found after surgery [17].

Transpedicular screw placement using the traditional freehand technique is mostly related to personal experience. The malposition rates differ between 21.1 and 39.8% (20–23). Learch et al. found screw malposition of 20% in the hands of experienced spine surgeons in a cadaver study. Three-dimensional CT guidance was found to be the best navigation device causing the least number of malposition but this is a time consuming and expensive method [18]. Kim et al. used this method and found 7.5% screw malpositions (5 screw malpositions in 66 cases) [19]. In some series, experienced spine surgeons reported similar malposition rates without the use of navigation systems [20, 21]. Kotil and Bilge reported similar rates of malposition [22].

They concluded that screw insertion without fluoroscopic guidance resulted in less operation time, less incidence of infection, and lower radiation exposure [22]. Laine et al. reported a malposition rate of 13.4% using the conventional technique and a rate of 7.1% using the computed tomography [23].

Kotil and Bilge applied 306 pedicle screws in the thoracolumbar area and reported 4 lateral walls, 7 upper endplate, 2 discs, and 6 medial wall violations. Two of these had nerve root irritations [22]. Castro et al. reported 49 perforations and 5-root lesions among 123 transpedicular screws [24]. Gertzbein and Robbins reported 48 malpositions and 2 minor neurological complications in 167 screws [25]. Guven et al. reported 38 (10%) malpositions in 379 screws [26].

It is not known whether the medial wall violations are done during or after pedicle cannulations or if it is possible to cause medial wall breaches after a correct cannulation of the pedicle. Also effect of the changes in the insertion angle after cannulation of the pedicle is not known. The advantage of our study is that it is done on human cadavers as animal cadavers cannot represent the anatomy correctly.

When we performed pedicle screw, we should measure all of the pedicles diameter by the computed tomography. This was limitation of our study.

In conclusion, we found that medial wall breaches can still happen after a correct cannulation of the pedicle. The change in insertion angle is one factor but we found that the most important factor is the use of a pushing force to insert the screw when there is a resistance. Resistance is a strong indicator that the screw leans on the medial pedicle wall and the insertion angle is wrong. When this is the case, the screw should be removed and the screw trajectory should be rechecked. The pedicle seems to be extremely tolerant to insertion angulation mistakes up to 10 degrees and tends to lead the screw into the correct path spontaneously. The screws without a self-tapping notch seem to be safer than notchless screws.

## Figures and Tables

**Table 1:** Pedicle complications within the groups.

Findings	Group 10 *N* (%)	Group 10P *N* (%)	Group 15 *N* (%)	Group 15P *N* (%)	Total *N* (% = *N*)
Medial wall fissure-fracture	0 (%0)	6 (%24)	1 (%4)	10 (%40)	17
Canal penetration of the screw	0 (%0)	0 (%0)	0 (%0)	12 (%48)	12
Leaning of the screw to the medial pedicle wall	0 (%0)	0 (%0)	8 (%32)	0 (%0)	8
Intact pedicle	25 (%100)	19 (%76)	16 (%64)	3 (%12)	63
Total number of pedicles	**25**	**25**	**25**	**25**	**100**

**Table 2:** Distribution of results in the “free drive” and the “push drive” groups.

Complications	Push drive *N* (%)	Free drive *N* (%)	Total *N* (*N* = %)
Pedicle fissure or wall defect	28 (%56)	1(%2)	29
Screw leaning	0 (%0)	8 (%16)	8
Intact pedicle	22 (%44)	41 (%82)	63
Total	**50**	**50**	**100**

**Table 3:** Distribution of results according to the type of screws.

Pedicle complication	Group 10		Group 10P		Group 15		Group 15P	
	B	N	B	N	B	N	B	N
Present	0	0	3	3	3	6	10	12
Absent	13	12	9	10	9	7	3	0

N: novel II (6.5 mm with notch), B: blackstone (6.0 mm without notch).
